# Reduced Susceptibility and Increased Resistance of Bacteria against Disinfectants: A Systematic Review

**DOI:** 10.3390/microorganisms9122550

**Published:** 2021-12-10

**Authors:** Urška Rozman, Marko Pušnik, Sergej Kmetec, Darja Duh, Sonja Šostar Turk

**Affiliations:** 1 Faculty of Health Sciences, University of Maribor, Žitna ulica 15, 2000 Maribor, Slovenia; pusnik.marco@gmail.com (M.P.); sergej.kmetec1@um.si (S.K.); sonja.sostar@um.si (S.Š.T.); 2 Chemicals Office of the Republic of Slovenia, Ajdovščina 4, 1000 Ljubljana, Slovenia; darja.duh99@gov.si

**Keywords:** antimicrobial resistance, susceptibility, disinfectants, bacteria

## Abstract

Disinfectants are used to reduce the concentration of pathogenic microorganisms to a safe level and help to prevent the transmission of infectious diseases. However, bacteria have a tremendous ability to respond to chemical stress caused by biocides, where overuse and improper use of disinfectants can be reflected in a reduced susceptibility of microorganisms. This review aims to describe whether mutations and thus decreased susceptibility to disinfectants occur in bacteria during disinfectant exposure. A systematic literature review following PRISMA guidelines was conducted with the databases PubMed, Science Direct and Web of Science. For the final analysis, 28 sources that remained of interest were included. Articles describing reduced susceptibility or the resistance of bacteria against seven different disinfectants were identified. The important deviation of the minimum inhibitory concentration was observed in multiple studies for disinfectants based on triclosan and chlorhexidine. A reduced susceptibility to disinfectants and potentially related problems with antibiotic resistance in clinically important bacterial strains are increasing. Since the use of disinfectants in the community is rising, it is clear that reasonable use of available and effective disinfectants is needed. It is necessary to develop and adopt strategies to control disinfectant resistance.

## 1. Introduction

Disinfectants, defined as biocides “Main group 1” [1], are an essential tool in combatting the spread of infectious diseases. When used properly and according to the instructions, disinfectants can help prevent pathogens’ transmission and spread, especially in nosocomial infections. With the rise of life-threatening infections with antibiotic-resistant bacteria and newly emerging viruses, the use of disinfectants and virucidal sanitizing agents has increased [2,3].

Disinfectants contain one or more biocidal active substances by which harmful organisms are chemically or biologically deterred, rendered harmless or destroyed [4]. In the healthcare sector, in addition to hand hygiene, the disinfection of surfaces is just as crucial to effectively protect patients, healthcare workers and visitors from the transmission of pathogens [5,6]. Biocidal products are also used in everyday hygiene, where consumers are offered a wide range of antibacterial cleaners, hygienic dishwashers, anti-sweat textiles, hygiene wipes and hand disinfectants. Most of them contain biocidal active substances in various amounts [7,8]. Although personal and household hygiene is often equated with antimicrobial products, regular handwashing without disinfectants is far more essential and sufficient [9]. However, overuse and improper use of disinfectants can accumulate to be reflected in disinfectant resistance, potentially changing our way of life, from compromising food security to threatening our healthcare systems [6].

Antimicrobial resistance has aroused great interest in the scientific and medical community in the case of antibiotics [10,11,12,13], but less interest has been paid to disinfectants, widely used, mainly in clinical settings [14,15]. Overuse and, more importantly, misuse of disinfectants may reduce the susceptibility of target organisms to clinically important antimicrobials due to cross-resistance and/or co-resistance mechanisms [16,17,18,19]. Therefore, it is necessary to pay more attention to the most broadly used disinfectants, i.e., human personal hygiene products, surface/material disinfectants, and algaecides [20,21]. It is advised to use comparable products without these biocidal agents to avoid unnecessary selection pressure of disinfectants on bacteria. Preferably, if a common handwashing agent can deactivate bacteria, there is an incentive to promote such use [22,23].

Decreased susceptibility or even resistance against disinfectants can occur due to various inside and outside cellular mechanisms resulting from bacterial phenotypic and genotypic adaptation [3]. Unlike antibiotics, the mode of action of disinfectants can be unspecific, targeting different processes or sites in bacterial cells and inflicting cell damage to multiple bacteria; hence resistance development is unlikely [24,25,26]. Nevertheless, bacterial resistance to disinfectants can be created by the mutation or amplification of an endogenous chromosomal gene, by acquiring resistant determinants on chromosomal genetic elements like plasmids, transposons, and integrons [23,27], or due to changes in cell envelope permeability, increased efflux pump expression and the specific mechanisms of phenotypic traits [28]. Another means of bacterial adaptation presents through disinfectant inactivation or neutralization [29] and biofilm formation.

Decreased susceptibility and acquired resistance to disinfectants has been documented [20,30,31,32], primarily against less reactive active ingredients, such as quaternary ammonium compounds (QAC), biguanides, phenols [2], benzalkonium chloride, triclosan (TCS; polychlorinated phenoxy phenol), chlorhexidine (CHX) [33], didecyl dimethyl ammonium chloride (DDAC) [34], tetracycline and chloramphenicol [35].

This review aims to describe whether mutations and thus decreased susceptibility occur in bacteria during long-term use and exposure to disinfectants.

## 2. Materials and Methods

### 2.1. Data Sources and Search Strategy

We performed a systematic review using the examination, analysis and synthesis of literature and the compilation method. We followed the PRISMA guidelines [36]. The search was performed using search terms in English: (susceptibility OR resistance) AND (disinfectants OR biocides) AND (bacteria OR microorganisms). A literature search was conducted with the databases PubMed, Science Direct and Web of Science. We used the following search limits: research papers published in English related to the research topic until June 2020. We used the same search terms, search limits, inclusion, and exclusion criteria in all the databases. Predetermined inclusion and exclusion criteria were applied as presented in Table 1.

### 2.2. Study Selection

**Figure 1:**
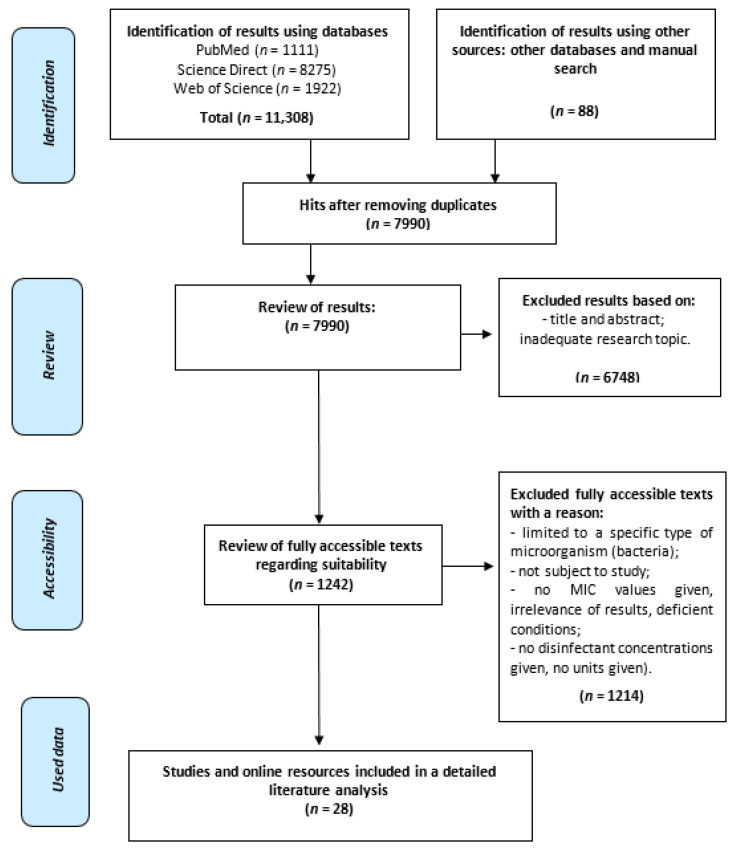
Flow diagram search strategy according to PRISMA recommendations.

After revision of the databases, the results (*n* = 11,308) were exported and compiled with Mendeley’s reference management software. We also performed hand searching and included 88 articles. Mendeley’s automated process removed duplicates (*n* = 3318), followed by a manual search to identify and remove additional duplicates. The authors screened all abstracts (*n* = 7990). The search focused on articles describing bacteria that developed a substantial decrease in disinfectant susceptibility with known biocide ingredients. There were multiple reasons for excluding studies, mostly for lacking MIC values or disinfectant concentrations. For the final analysis and the review, 28 sources that remained of interest were included and screened based on their full text by two independent reviewers (See Figure 1).

### 2.3. Data Extraction and Analysis

The relevant data were first extracted by MP and checked by UR. The characteristics of the identified relevant sources were presented in a table, where we described the findings from the review and analysis of the relevant literature. The main findings from the identified sources were highlighted. The extracted data included: (1) disinfectant category; (2) publication author(s), year, country, journal; (3) study aim/purpose; (4) main results of the identified research study.

## 3. Results

Articles describing the resistance of bacteria against seven different disinfectants were identified, namely: triclosan (10 articles), peracetic acid (2 articles), hydrogen peroxide (3 articles), ethanol and isopropanol (1 article), formaldehyde and glutaraldehyde (2 articles), chlorhexidine (4 articles), benzalkonium chloride and didecyldimonium chloride (5 articles). For this review, the definition of bacterial resistance to a disinfectant is based on an importantly decreased susceptibility in different tests (e.g., disk diffusion test, minimum inhibitory concentration MIC) reported by the clinical microbiology or research laboratories described in the reviewed studies. All 28 selected publications that met the search criteria are classified in Table 2. 

Multiple studies have shown an increased MIC from approximately 4- to 60-fold for specific bacteria for the disinfectant triclosan, making it epidemiologically relevant for increased bacterial adaptability and resistance [16,37,38,39,40,41,42,43,44,45]. The mechanisms for the elevated MIC were various mutations at the genetic level. For *E. coli* these were: deletion of the *ycjD* gene [45], mutation at codon 93 of the *fabI* gene, and mutation of the MarR transcription activator within the *marRAB* operon c, which regulates the operation of efflux pumps [41,43]. For *P. aeruginosa*, there was deletion of the *fabV* gene, leading to a decreased fatty acid synthesis and consequent inhibition of the production of acyl-homoserine lactones and other virulence factors, such as LasA/LasB, alkaline proteases, phospholipases, lipases, exotoxin A, rhamnolipid and pyocyanin, and a reduced pathogenicity [46]. It also affects the *MexCDOprJ* gene, PAO1, which encodes 12 RND pumps [47,48]. For *S. aureus*, intracellular malonyl-CoA inhibits the activity of the transcriptional repressor FapR, which directly interacts with the *fabI* gene, physiologically regulating its expression. This results in the most common mutations, polymorphisms, within the coding regions of C34T and MO035 in the sa-FabI region [49,50]. For *S. enterica* there was: a mutation in the *fabI* gene, mutation of the *AcrAB* and *TolC* genes that regulate efflux pumps, and inactivation of the transcriptional regulators ramA and marA [51,52,53].

For the disinfectant peracetic acid, the MIC was raised four-fold in one of the three bacteria tested (*P. aeruginosa*), while in the others, there were no significant changes in susceptibility. According to the described example, it could be classified as relevant in the indication of resistance, although the results are currently deficient due to the lack of multiple studies and unequal conditions [54,55]. For *E. coli*, mutations in the genes *erm (B)*, *tet (M)*, and *tet (L)* were observed [56].

For the disinfectant hydrogen peroxide, the MIC was also relevantly elevated in only one of the three bacteria studied (*A. baumannii*) and can be treated as a possible indicator of resistance here as well. However, due to deficient studies and unequal conditions, no conclusion regarding resistance can be made [57,58,59]. The cause of the elevated MIC were gene mutations. In all bacteria, mutations were in genes that regulate catalase (Kat), alkyl hydroperoxide reductase, and DNA-binding proteins that allow the catalase-reversible mechanism’s inhibitory effect on SpxB expression [60,61].

For the disinfectant chlorhexidine, the MIC has risen by almost 32–150 times in multiple relevant studies reviewed, making it epidemiologically relevant for increased bacterial adaptability and resistance, and a research/clinically relevant biocide [62,63,64,65,66]. The causes of the elevated MICs were gene mutations. For *Enterococcus,* mutation of the *efrA* and *efrB* genes that alter the expression of the EfrAB efflux pump of the ABC family, and hydrophobicity of the bacterial surface were observed [67,68]. For *S. aureus* and MRSA, mutations of *qacA*, *qacB*, *smr* and *norA* genes were observed [69,70,71,72]. For *P. aeruginosa*, mutation of efflux pump genes, such as *MexCD-OprJ* and *oprH-phoPQ* initiated by the stress response factor AlgU [69,70,71,72,73,74], and a decreased regulation of genes that encode proteins involved in membrane transport, oxidative phosphorylation, electron transport, and DNA repair were observed [75,76,77].

For the disinfectant benzalkonium chloride in multiple relevant studies reviewed, in three of the four bacteria, the MIC rises only 1–4-fold. This could make it epidemiologically relevant for increased bacterial adaptability and resistance, and a research/clinically relevant biocide [68,78,79,80,81]. The cause of the elevated MIC were gene mutations. For *P. aeruginosa*, mutations of efflux pump genes such as MDR *mexA-mexB-oprM* and *mexC-mexD-oprJ* were observed [82,83]. For *E. coli*, a mutation in the *sugE* gene located in the 94 regions of a chromosome that phenotypically inhibits a *groEL* mutation were observed [84,85]. For *S. aureus*, mutations of six different genes (i.e., *qacA / B*, *qacC (smr)*, *qacG*, *qacH* and *qacJ*) that contribute to the development of resistance to QAC were observed [86,87,88,89].

For disinfectants containing alcohols, aldehydes and iodine compounds, no relevant changes of MIC values were reported [90,91,92,93,94].

The MIC values for most commonly used biocides against clinically important bacteria are presented in Table 3. The bacteria considered resistant had an increased MIC at least two times the average MIC in the first column.

## 4. Discussion

### 4.1. Criteria to Identify Resistant Strains

In order to understand resistance, there is an emphasis to distinguish between intrinsic and extrinsic resistance. Inherent resistance, known as natural resistance, is chromosomally encoded resistance, which determines the basic spectrum of effects of a disinfectant and the phenotypic resistance, e.g., biofilms. Extrinsic or acquired resistance develops through mutation by incorporating mobile genetic elements (horizontal gene transfer), transferable plasmids and other cell elements [114,115]. A clear distinction also needs to be made between phenotypic adaptation, which is reversible when exposure to the biocides ends, and acquired resistance, being genetically determined and usually stable [115]. When studying antibiotic resistance, the European Committee on Antimicrobial Susceptibility Testing has decided to: “define separate dividing points for the detection of bacteria with resistance mechanisms and the monitoring of resistance development using wild-type cut-off values (WCV) or epidemiological cut-off values (ECOFF or ECV) and the guidance of therapy via clinical breakpoints” [101,116]. As defined by the European Committee on Antimicrobial Susceptibility Testing, the ecological concept of antibiotic resistance states that ECOFFs are defined based on the normal distribution of MICs in a given bacterial species. Any isolate with a MIC above the epidemiological cut-off value (ECOFF), which is the upper limit of the normal distribution of the MIC for a given antimicrobial agent and a particular species, is considered resistant [43,96].

In the case of studying biocide resistance, however, no limits have been set so far, and there are no clear criteria to determine whether a microbe is susceptible to the biocide or not. Therefore, we can use the average MIC values obtained from individual laboratory studies conducted under relatively similar conditions. The observed relevant increase in the MIC value can indicate a decreased susceptibility or even resistance. When interpreting the results, the in-use concentration of the disinfectants used must be considered since the in-use concentration may also be higher than the actual measured MIC values. In this case, we cannot talk about the resistance but only about a decreased susceptibility.

### 4.2. Most Common Bacterial Mechanisms to Develop Resistance against Disinfectants

Bacteria control and overcome the effect of disinfectants in different ways (Table 4), such as restricted permeability of the cell wall, the expression of efflux systems, enzymatic degradation, changes in target sites, and the formation of biofilms [23,117]. Changes in cell surface hydrophobicity, ultrastructure, protein composition, and fatty acid modifications appear to occur [118,119]. For example, inactivation of the lipooligosaccharide biosynthesis genes causes resistance in *A. baumannii* [120]. Modifying the outer membrane proteins and an increased expression of cellular structures may increase the sensitivity to disinfectants [118,119,121]. Impermeability of the outer membrane occurs because of the lipopolysaccharide component, which increases the penetration of disinfectants and affects the size and expression of pores, thereby preventing entry and affecting sensitivity [122]. The hydrophilic porin channels on the outer membrane regulate the passage of solutes and are a significant barrier to hydrophilic substance penetration [123]. They also have a negative charge, which can cause the disinfectant molecules to bounce away from the bacterial cell.

Bacteria can also grow as biofilms, endospores, and within cellular macrophages. In most natural habitats, microorganisms grow and survive as associated biofilms [124]. Monocultures of several different species or mixed phenotypes of a particular species can form biofilms. It is a community of nonmobile microorganisms that are irreversibly attached to a surface and inserted into a polymeric extracellular matrix. The insensitivity of biofilms to disinfectants is due to altered microbial growth rates, which can be attributed to nutrient depletion in the biofilm, and disinfectant binding to the biofilm, which is neutralized or degraded [35]. Such an organization may moderate the concentration of antimicrobial disinfectants and antibiotics to which deeper biofilm cells are exposed. Such cells accidentally grow slowly, starve, and express stress phenotypes, including regulating efflux pumps and flushing out disinfectants [125].

Slightly less effective mechanisms involve the enzymatic degradation or inactivation of disinfectants when concentrations of agents, such as formaldehyde, chlorhexidine, and quaternary ammonium compounds, are lower than those used in clinical trials practice [126]. The exposure of bacteria to minimal inhibitory concentrations of disinfectants results in the induced expression of neutralizing enzymes, which is crucial for the biodegradation of disinfectants [127]. Examples of the neutralization of disinfectants have been given in several species of bacteria, for example, *Pseudomonas fluorescens* TN4 isolated from sludge was able to degrade DDAC, which belongs to the group of quaternary ammonium compounds. The isolate was also able to degrade other QACs by the N-dealkylation process [128].

One major cause of bacterial resistance is the active transport of substances to the cell exterior, the so-called efflux with proteins. Efflux pump mechanisms perform essential physiological functions [97]. Although existing in all living cells, those found in bacterial and mammalian cells are especially important for clinicians and pharmacologists since they constitute an important cause of antimicrobial resistance. Multidrug Resistance (MDR) efflux pumps present an ongoing research topic in antibiotic resistance and are also responsible for disinfectant resistance mechanisms [129]. One of the fundamental mechanisms of action is the efflux pump’s influence and the modulation of its genes. These efflux systems existed in bacteria long before the use of disinfectants and antibiotics in humans to treat infections. The mechanism involves the secretion of toxic compounds through a bacterial cell wall with a membrane-bound protein composed of at least three components. The increased expression of these pumps can raise the minimum inhibitory concentration to a high level, resulting in resistance to disinfectants [130] and greater sensitivity and cross-resistance to antibiotics [40]. Research data show that pump expression reduces the efficacy of various classes of disinfectants, including chlorhexidine digluconate, hydrogen peroxide, benzalkonium chloride, chloroxylenol, iodine compounds, triclosan, quaternary ammonium compounds, phenolic parabens and intercalates [131,132]. Among the best-studied systems of genes that regulate the secretion of biocides are *mexAB-oprM*, *mexCD-oprJ* and *mexEF-oprN* in *P. aeruginosa* [133], *acrAB-tolC*, *acrEF-tolC* and *emrE* in *E. coli* [134], *smeDEF* in bacteria *Stenotrophomonas maltophilia* [135], and *norA* and *mepA* in *S. aureus*. In the highly resistant nosocomial bacterium *A. baumannii*, the efflux activity is regulated by the *quacA* and *quacB* genes [57,77,136]. Bacteria use the same pumps to remove antibiotics and biocides. Thus, they can select antibiotic-resistant mutants that over-regulate such pumps [137].

Another important factor contributing to the development of disinfectant resistance is the mode of action of disinfectants. Biocides have a broader spectrum of activity and may have multiple targets, while antibiotics tend to have specific intracellular targets [29]. However, in the case of biocides with a particular antimicrobial mechanism (e.g., quaternary ammonium compounds—QAC’s), the development of antimicrobial resistance against disinfectants, and cross-resistance to antibiotics, are especially well documented [138].

**Table 4:** Disinfectant and bacterial mechanisms for the most commonly used disinfectants.

Disinfectant Category	Active Ingredient	Use in Clinical Setting	Disinfectant Working Mechanism	Bacterial Adaptation to Disinfectant	Ref.
Alcohol	Ethic Alcohol (Ethanol)	70–95% Ethanol solution	Denaturation of bacterial membrane proteins and dissolving lipid components such as antiparallel β and 3_10_ helical turns of proteins, C-H deformations in lipids, inhibition of nutrient transport via membrane-bound ATPases, alteration of membrane pH and membrane potential.	Horizontal gene transfer, transformation and transduction and core genome mutations in the chromosome nucleotide position on the *rpoB* gene β subunit of RNA polymerase.	Alcohol working mechanism: [6,139,140,141] Alcohol adaptation: [91,142]
Aldehydes	Formaldehyde	5% Formaldehyde solution	Cross-linking of protein’s free amino groups and inhibition of transport processes, RNA, and DNA.	Inactivation of formaldehyde through a metabolic system dependent on pterin cofactors, sugar phosphates, and those dependent on glutathione. Three separate enzymes catalyze successive stages of formaldehyde oxidation to CO_2_. These are the enzyme Gfa, alcohol dehydrogenase, and thioesterase. The specifically transmissible plasmid *adhC* gene encodes a glutathione-dependent formaldehyde dehydrogenase that causes inactivation.	Formaldehyde working mechanism: [3,29] Formaldehyde adaptation: [143,144,145]
Biguanides	Chlorhexidine (gluconate/diacetate)	0.5% Alcohol solution (70%)	Inhibition of cytoplasmic membrane function and membrane-bound enzymes and leakage of intracellular components; inhibitor of both membrane-bound and soluble ATPase as well as of net K+ uptake, also collapses the membrane potential and has the potential for ATPase inactivation.	Induced gene expression of efflux pumps with upregulation and downregulation of coding genes (for an MFS transporter and HlyD-like periplasmic adaptor protein), active ingredient inactivation and alteration of the cell wall, increase in cell envelope components such as lipopolysaccharide or phospholipid caused by progressive mutations.	Chlorhexidine working mechanism: [29,33] Chlorhexidine adaptation: [29,33,43,65,146]
Bisphenol	Triclosan		Inhibition of enoyl-acyl carrier protein (ACP) reductase (FabI enzyme) in *E. coli*, *P. aeruginosa*, *S. aureus* and its homologue InhA in *M. smegmatis*, *M. tuberculosis*.	Target mutations, increased target expression (overexpressed genes *mufA1* and *mufM*), active cell excretion, enzyme inactivation/ degradation. Increased concentration of branched chain fatty acids in the cell membrane occurs and multiple amino acids are changed in the *fabI* gene along with an increased concentration of the FabI protein through heterologous duplication and increased activity of ENR isoenzymes.	Triclosan working mechanism: [19,39,134,147,148] Triclosan adaptation: [30,149,150,151]
Halogen releasing agents	Povidone-Iodine	1–10% Iodine solution	Intracytoplasmic protein oxidation (cysteine and methionine), nucleotide and fatty acid function disruption, inhibition of production and release of bacterial exotoxins such as α-hemolysin, phospholipase C and enzymes such as elastase and β-glucuronide.	Formation of a biofilm and thickening of the cell wall.	Halogen releasing agents working mechanism: [29,152,153] Halogen releasing agents adaptation: [153,154]
Peroxygens	Hydrogen Peroxide	3–6% Hydrogen Peroxide	H_2_O_2_ acts as an oxidant by producing hydroxyl or ferryl free radicals which disrupt the function of lipids, proteins-sulfhydryl (SH) and sulfur (SS) bonds and DNA.	Gene *katA* role of catalase, and peroxidase enzymes which neutralize H_2_O_2_. Bacterial cells form thick biofilm formations.	Hydrogen peroxide agents working mechanism: [29,155] Hydrogen peroxide agents adaptation: [155,156,157]
Quaternary Ammonium Compounds	Benzalkonium Chloride	0.01–5% Benzalkonium chloride	Cationic amphiphilic properties destabilize the pathogen’s surface by forming electrostatic interactions with negatively charged components. Cytoplasmic membrane damage of phospholipid components occurs, distortion and protoplast lysis occur under osmotic stress. Leakage of low molecular weight components and eventual cell wall lysis.	Downregulation of membrane porins, overexpression or modification of efflux pumps (Mrdl EmrE MdfA) with mutations of the Mex system, horizontal gene transfer of transposon elements (Tn6188) and stress factors, biofilm formation, and biodegradation by dealkylation.	Benzalkonium chloride releasing agents working mechanism: [29,78,158] Benzalkonium chloride releasing agents adaptation: [82,159]

## 5. Conclusions

Antimicrobial resistance in healthcare facilities has been occurring and regularly increasing over the last ten years. Growing evidence from in vitro studies has shown that bacteria have a tremendous ability to respond to chemical stress caused by biocides by several different mechanisms [160]. The main reason for emerging resistance is attributed mainly to the overuse, abuse and misuse of disinfectants [160,161,162]. Relevant increases in MIC concentrations, changes at the genetic level, and clearly altered mechanisms were observed in studies of several bacterial species in the presence of disinfectants. Through the most relevant of the reviewed articles, we can define the results for disinfectants based on triclosan and chlorhexidine, where the critical deviation of the MIC was observed in multiple studies.

Given the ongoing problems with multiple antibiotic resistance in clinically important bacteria strains and the potential for increased resistance to disinfectants, the use of which is rising in the community, it is clear that the prudent use of available and effective antimicrobials is needed. It is essential to develop and adopt strategies to control disinfectant resistance, for which the following factors will make a significant contribution. To solve the disinfectant resistance problem, it is essential to comprehensively summarize the disinfectant resistance mechanisms and to understand the resistance influencing factors [163]. It is also necessary to establish ECOFF values for biocides, without which any research is challenging and, to some extent, inaccurate. Harmonized methods for biocide susceptibility testing need to be developed.

Further studies are needed to establish a link between disinfectant exposure and resistance development, as many studies in clinical or external settings are currently limited. The rotation of disinfectants, where one disinfectant should be replaced by another having a different mechanism of action, is recommended [164]. The same types of disinfectants are used both in healthcare institutions and among the general population; therefore, their prudent use and consumption, as we know in the case of antibiotics, are complicated to control. Since selective pressure caused by disinfectants is exerted on both commensal and pathogenic bacteria [165,166], monitoring for resistant genes in nonpathogenic or commensal bacteria would make sense. Health-related infections acquired in the community need to be researched annually. More attention should be paid to the correct use of disinfectants by the general public, although supervising the proper use of disinfectants among the general population is very difficult to implement.

The risks and benefits of using disinfectants in the environment need to be weighed to determine whether additional precautions are required to guide the development and use of disinfectants [167]. If bacterial resistance increases and develops against many regularly used disinfectants in clinical and industrial settings, overuse in reflection of the COVID-19 pandemic could place an additional burden on global public health [168].

## Figures and Tables

**Table 1:** Research strategy inclusion and exclusion criteria.

Databases:	PubMed, Science Direct and Web of Science	
	Inclusion Criteria	Exclusion Criteria
Problem:	Bacteria that developed a substantial decrease in susceptibility to disinfectants with known biocide ingredients	Bacteria that did not develop a substantial decrease in susceptibility to disinfectants with known biocide ingredients
Intervention/ Treatment:	Susceptibility test (disk diffusion test, minimum inhibitory concentration)	Susceptibility test results not included
Outcome:	Reduced susceptibility and increased resistance of bacteria against disinfectants	Bacteria without reduced susceptibility and increased resistance against disinfectants
Types of research:	Research article experimental and quasi-experimental	Systematic review articles or other types of reviews Duplicates, commentaries, editorials, conferences, and research protocols
Search limits		
Timeframe:	Until December 2020	
Language:	English	

**Table 2:** Study characteristics of identified research studies about disinfectant resistance.

Author, Year, Country, Journal	Study Aim/Purpose	Main Results of Identified Research Studies
Triclosan		
Cottell, et al. (2009), Great Britain and Germany, Journal of Hospital Infection	Determining the minimum inhibitory concentrations of triclosan with broth- and agar-dilution methods. Using the British Society for Antimicrobial Chemotherapy guidelines, the antibiotic susceptibilities were determined. Exploring further linkages between triclosan exposure and the emergence or lack of bacterial antibiotic resistance.	Triclosan MICs were significantly higher for the mutant strains *S. aureus* T3 and *E. coli* TM3 compared with the parent strains *E. coli* ATCC 8739 and *S. aureus* NCIMB 9518. Significantly higher MIC was also observed in the triclosan-tolerant strain *A. johnsonii* RC compared to the sensitive counterpart.
Byung, et al. (2010), Great Britain, Journal of Antimicrobial Therapy	Studying defense mechanisms against triclosan in mutants derived from the *E. coli* strain, which carry the target triclosan-resistant enzyme, FabI (G93V).	The MIC of triclosan for the *E. coli imp4231* FabI (G93V) mutant with different concentrations after 20 h of exposure resulted in 8 mg/L. This is approximately 400 times higher when compared with the parent strain, where the MIC was 0.02 mg /L. The highest MIC of triclosan (80 mg/L) was in *E. coli* IFN4.
Cameron, et al. (2019), India, PLoS One	Identifying the functional mechanisms of triclosan resistance in waste waters metagenomes and assessing the frequency of resistance in clinical isolates of *E. coli* and *Enterococcus* spp.	Three *E. coli* isolates had up to 64-fold higher triclosan MICs (2 to 8 mg/L) than the original MICs and were presumed ESBL producers.
D’Arezzo, et al. (2012), Italia, BMC Research Notes	Evaluating the antimicrobial activity of triclosan and chlorhexidine digluconate (also for two commercial disinfectants) against the epidemic strain of *P. aeruginosa*.	An extremely high level of triclosan resistance (MIC 2125 mg/L) was observed for the *P. aeruginosa* epidemic strain. The same strain was susceptible to chlorhexidine digluconate (MIC 12.5 mg/L). The epidemic strain survived for more than 120 h in the presence of 3400 mg/L (≈ 1.6 × MIC) of triclosan upon gradual adaptation.
Huang, et al. (2016), China, Frontiers of Microbiology	Demonstrating the functional characteristic of triclosan-resistant enoyl-acyl protein reductase carrier (FabV) in *P. aeruginosa.*	The plasmid-bearing strain *P. aeruginosa* PAO170 (1fabV) (pSRK-PI and pSRK-PV) was triclosan resistant; the triclosan MIC was above 2000 μg/mL.
Forbes, et al. (2015), Great Britain, Antimicrobial Agents and Chemotherapy	Antimicrobial triclosan susceptibility testing, cell mobility and morphology in small colonies of *Staphylococci.*	*S. aureus* produced small colony variants with decreased triclosan susceptibility (4/6 test bacteria) when exposed to triclosan. The susceptibility of strains P0 to P10 increased in MIC (4- to 31-fold) and MBC increased from 3- to 16-fold. ATCC 6538 R1 had a MIC of up to 31 mg/L.
Bayston, et al. (2007), Great Britain, Journal of Antimicrobial Chemotherapy	Determining the antimicrobial activity duration of triclosan-impregnated silicone. Reporting about the development of MRSA resistance during experimental exposure.	Two of the three MRSA strains showed impaired coagulase production and decreased deoxyribonuclease production. Triclosan MICs increased between 8- and 67-fold (MIC up to 4.0 mg/L).
Copitch, et al. (2010), Great Britain, International Journal of Antimicrobial Agents	Determining the reduced susceptibility to triclosan in the group of *S. enterica* isolates and to identify and describe the mechanisms of resistance.	The level of resistance to triclosan was generally low in *S. enterica* isolates (triclosan range MIC 0.25–4 mg/L). Increased efflux activity was observed in multidrug-resistant and triclosan-resistant strains when comparing them to the strains with a reduced susceptibility to triclosan alone.
Condell, et al. (2012), Ireland, Journal of Proteomics	In the study, the authors compared the proteomic profile of the susceptible serovar *S. enterica* Typhimurium with its isogenic triclosan tolerant strain to decode cellular mechanisms that promote biocide tolerance.	Changes in the proteome of *Salmonella* were observed when exposed to sublethal concentrations of triclosan, which gave insights into mechanisms for the response and tolerance.
Webber, et al. (2008), Great Britain, Journal of Antimicrobial Chemotherapy	The purpose of the study was to describe the mechanisms of triclosan resistance in *S. enterica* Typhimurium.	Three triclosan-resistant phenotypes were classified as low MIC (MIC <8 mg/L), medium MIC (MIC 16–32 mg/L), and high MIC mutants (MIC > 32 mg/L). The most resistant mutant was strain L702 with MIC 64 mg/L.
Peracetic acid		
Van der Veen and Abee (2011), Netherlands, International Journal of Food Microbiology	Studying single and mixed-species biofilm formation (*Listeria monocytogenes* EGD-e and LR-991), with *Lactobacillus plantarum* WCFS1 as the secondary species. Determining resistance to benzalkonium chloride and peracetic acid.	Resistance to benzalkonium chloride is higher in single and mixed-species biofilms than in planktonic grown cells. After exposure for 15 min to 100 μg/mL, mixed-species biofilms are more resistant to benzalkonium chloride than single-species biofilms. The resistance against peracetic acid treatments (15 min to 100 μg/mL) is also higher in single and mixed-species biofilms than planktonic grown cells, but the differences are less pronounced.
Spoering and Lewis (2001), USA, Journal of Bacteriology	Studying biofilms of *P. aeruginosa* wild-type strain PAO1 and comparing its resistance against biocide when comparing it to planktonic cells.	When comparing biofilms to logarithmic-phase planktonic cells, biofilms were considerably tolerant to the biocide. On the other hand, stationary-phase planktonic cells were more tolerant of peracetic acid than biofilms. The MBC for all three populations was 400 g/mL.
Hydrogen Peroxide		
Pericone, et al. (2000), USA, Infection and Immunology	To determine if *Streptococcus pneumoniae* and *Haemophilus influenza* produce substances (hydrogen peroxide) that inhibit the other bacteria’s growth.	*H. influenzae* was most susceptible to growth inhibition, and killing by H_2_O_2_ (MIC, 0.4 mM; MBC, 0.5 mM). *N. meningitides* was also relatively sensitive (MIC, 0.4 mM; MBC, 5.0 mM). *M. catarrhalis* was shown to be relatively insensitive (MIC, 1.1 mM; MBC, 160 mM) like *Pneumococcus* (MIC, 1.6 mM; MBC, 80 mM), which could explain the ability to survive endogenously produced hydrogen peroxide.
Wesgate, et al. (2016), Great Britain, American Journal of Infection Control	Exposure of *Staphylococcus aureus* and *Escherichia coli* to triclosan, chlorhexidine gluconate solution, hydrogen peroxide and a hydrogen peroxide-based product. Determination of the susceptibility by combining standard efficacy protocols.	Significant increases in antimicrobial insusceptibility (MIC 69-fold; MBC 74-fold increase) were observed when exposing *S. aureus* for 5 min to 0.0004% triclosan.). A more than 30-fold increase in MIC was observed after *E. coli* exposure to bisphenol. No changes in the susceptibility profile (less than two-fold) were observed when exposing bacteria to 0.00005% CHG, 0.001% H_2_O_2_ and 0.001% Oxy BAC.
Lin et al. (2017), China, Frontiers of Microbiology	Using a PCR reverse transcriptase method for the assessment of the association between efflux pump gene expression and a reduced sensitivity for triclosan, chlorhexidine, benzalkonium chloride, hydrogen peroxide and ethanol.	A variety of susceptibilities to biocides was observed by the tested isolates. MICs for triclosan ranged from 2–256 μg/mL, for chlorhexidine 8–128 μg/mL, for benzalkonium bromide 4–32 μg/mL, (1.6–13 mg/mL), for hydrogen peroxide 47–376 mM, and for ethanol (60–180 mg/mL) at 7.5–22.5% (vol/vol).
Ethanol and Isopropanol		
Pidot, et al. (2018), Australia, Science Translational Medicine	In this study, the authors sought to compare the alcohol tolerance of 139 nosocomial *E. faecium* isolates obtained between 1997 and 2015.	Newer clinical isolates of *E. faecium* were more resistant to alcohol than their predecessors. Using a 70% isopropanol surface disinfectant, mutated *E. faecium* isolates were ten times more tolerant to disinfectant than isolates from decades ago. Strain ST796 had a reduced tolerance to isopropanol of 1.14 log^10^. Four hundred nucleotide positions mutated on two or more pairs of sequences.
Formaldehyde and Glutaraldehyde		
Gradel, et al. (2005), Denmark, Veterinary Microbiology	The study’s main objective was to perform a preliminary examination to detect apparent differences between *Salmonella* serotypes and isolates, to link them with the resistance to disinfectants, for which there are extensive data regarding Danish fattening flocks.	In MICs of five disinfectants commonly used in the Danish or English poultry sector, few variations were observed. Most differences from the isolates having high MICs were determined when using formaldehyde, but only a few isolates differed from the high MIC isolates when using the other four disinfectants.
Tschudin-Sutter, et al. (2011), Switzerland, Infection Control and Hospital Epidemiology	Determining the effectiveness of the endoscope cleaning procedure with glutaraldehyde in a Danish hospital against *P. aeruginosa* strains.	In samples obtained by endoscopes, *P. aeruginosa* was detected. During the disinfection procedure, a disinfectant based on glutaraldehyde showed no activity against two *Pseudomonas* outbreak strains when used under standard conditions at the recommended concentration. After reviewing the medical chart, six patients with circulatory and lower respiratory infections had an epidemiological link to the *Pseudomonas* outbreak strain. The most resistant strain needed the use of concentrations almost three times higher to achieve the same microbicidal effects.
Chlorhexidine		
Braga, et al. (2013), Portugal, Veterinary Microbiology	The isolation of *Enterococcal* strains from dust samples collected from Portuguese breeding pig establishments. Determining the sensitivity of strains to chlorhexidine, benzalkonium chloride, biguanide and QAC was studied. The presence of VRE in these samples was also investigated.	The maximum MIC value for benzalkonium chloride and chlorhexidine in VRE isolates was 4 µg/mL. This was also the highest MIC value for all the other 41 isolates. The exceptions were two vancomycin-intermediate isolates, with a MIC to chlorhexidine of 8 µg/mL.
Valenzuela, et al. (2013), Spain, Journal of Food Protection	*Enterococci* were isolated from different animal species and plant food wildflowers, animal infestations and clinical specimens. Determinations were made of the resistance incidence for biocides (quaternary ammonium compounds, bisphenol, biguanide) and copper sulphate.	Triclosan (250 mg/L) inhibited 98.16% of isolates. The greatest variability was observed for chlorhexidine (MICs from 2.5 to 2500 mg/L). For the inhibition of 74.57% of isolates from clinical samples, the required dose of chlorhexidine was 2500 mg/L. Inhibition of *Enterococci* by copper sulfate was observed in the range 4–16 mM.
Akinkunmi and Lamikanra (2012), Nigeria, Journal of Infection in Developing Countries	Examination of MRSA resistance to commonly used antibiotics and antiseptics in fecal sample isolates from children in the community	Among MRSA isolates, 68.8%, 75.0%, and 100% were more resistant to benzalkonium chloride, chlorhexidine, and cetrimide than *S. aureus* NCTC 6571. Among the methicillin-susceptible *S. aureus* isolates, 32.0%, 28.0%, and 56.0% were more resistant to benzalkonium chloride, chlorhexidine gluconate, and cetrimide than *S. aureus* NCTC 6571. MIC_50_ values for *S. aureus* were 8 mg/mL, 4 mg/mL and 32 mg/mL for benzalkonium chloride, chlorhexidine and cetrimide, respectively.
Thomas, et al. (2000), Great Britain, Journal of Hospital Infection	The aim was to investigate the effects of sub-MIC concentrations of CHX on gram-negative bacteria, particularly the *P. aeruginosa* strain, which is known to have an intrinsic resistance to CHX, and the susceptibility of CHX-resistant strains to antibiotics.	After the fourth subculture, growth occurred within 24 h with a further increase in the MIC in *P. aeruginosa* strains NCIMB 10421; the MIC was significantly increased from the original 10 µg/mL to more than 70 µg/mL. The significance of these findings is still unclear, as the concentration of CHX in clinical use is much higher than that at which the authors obtained resistance.
Benzalkonium chloride and didecyldimonium chloride		
Nasr, et al. (2018), Egypt, American Journal of Infection Control	The study aimed to evaluate the effect of isolated *Pseudomonas* exposure, the susceptibility to antibiotics, and to subinhibitory concentrations of two disinfectants, didecyldimonium chloride and sodium hypochlorite.	The development of antibiotic and biocidal resistant *Pseudomonas* strains can occur when using concentrations of sodium hypochlorite and didecyldimonium chloride. This study emphasizes the need for strict adherence to standard hospital disinfection policies to achieve adequate prevention and control of healthcare-associated infections. The MICs for all isolates ranged from 0.01% to 0.02% for sodium hypochlorite and 0.012% for didecyldimonium chloride.
McCay, et al. (2010), Ireland, Microbiology	Determining whether exposure of the population of *P. aeruginosa* (NCIMB 10421) to higher BAC levels in long-term continuous culture would result in a cross-adaptation to antimicrobials.	A method to enrich a continuous culture of *P. aeruginosa* NCIMB 10421, the MIC 25 mg/L of benzalkonium chloride was added (D = 0.04 h–1.792 h). The derivative PA-29 (696 h) showed a >12-fold reduced susceptibility to the biocide, MIC > 350 mg/L.
Fazlara and Ekhtelat (2012), Iran, American-Eurasian Journal of Agriculture	Evaluation of the antibacterial effects of benzalkonium chloride commonly used in the food industry, on six major food-borne pathogens.	The benzalkonium chloride MIC and MBC ranged between 40 and 45 mg/L for *E. coli*. The most susceptible and resistant bacteria were *L. monocytogenes* and *B. cereus* (MIC 30 and 35 mg/L and MBC 140 and 160 mg/L, respectively)
He, et al. (2014), China, Journal of Medical Microbiology	Studying isolates of BAC-resistant *Staphylococci* from the community environment with isolation, identification, and detection of BAC resistance genes.	The analysis of resistance genes showed that 41 strains contained qacA/B, 30 strains qacC, 25 strains qacG, 16 strains qacH, and eight strains qacJ. Because the BAC biocide affects these genes, this indicates an associated resistance in *Staphylococci.* The maximum MIC value for 63 strains of *S. aureus* ranged up to 32 μg/mL for BAC.
Yu, et al. (2018), China, Frontiers in Microbiology	The study examined the effect of BAC adaptation on antimicrobial susceptibility and tolerance to environmental loads and the role of efflux pumps in the adaptation of *L. monocytogenes.*	In BAC adapted *Listeria* strains, 18 EtBr strains had a MIC of 200 μg/mL, in 5 strains the MIC was > 200 μg/mL and in 2 strains it was 100 μg/mL.
Ramzi, et al., (2020), Morocco, BioMed Research International	Studying the antibacterial activity of quaternary ammoniums synthetic disinfectants for hospital environment isolates: *Escherichia coli*, *Klebsiella pneumoniae*, *Enterobacter cloacae*, *Pseudomonas aeruginosa*, *Acinetobacter baumannii*, and *Staphylococcus aureus*.	The tested disinfectant demonstrated an antibacterial effect against *S. aureus* and *S. aureus* ATCC 29213 (MIC of 0.25 mg/mL); the disinfectant spray showed effects in *E. coli*, *S. aureus*, *E. coli* ATCC 25922, and *P. aeruginosa* ATCC 27853 (MIC of 4 mg/mL) and *S. aureus* ATCC 29213 (MIC 2 mg/mL). Phagosurf ND^®^ inhibited the growth of *S. aureus* ATCC 29213 (MIC of 4 mg/mL).

**Table 3:** MIC of biocides and the expressed susceptibility/resistance of bacteria to disinfectants.

Bacteria/Disinfectant	MIC—Average Cut Off Values from Studies (mg/L) (Number of Strains)	MIC—Outstanding Elevated Values (mg/L) (Strain type)	Study Data Quality and Limitations	Main Results from Identified Studies—Resistance Relevance/Rise of MIC
Triclosan (0.5–2%) [95]				
*Escherichia coli*	2 mg/L (368 strains) [96]	8 mg/L (*E. coli* imp4231 FabI (G93V)) [81]	/	Relevant MIC increase indicates resistance.
*Escherichia coli*	2 mg/L (368 strains) [96]	8 mg/L (multiple strains) [38]	/	Relevant MIC increase indicates resistance.
*Escherichia coli*	2 mg/L (368 strains) [96]	1000 mg/L (*E. coli* TM3) [41]	Lack of precise evidence, unclear standards and research conditions.	Relevant MIC increase does not indicate resistance due to low-quality study.
*Pseudomonas aeruginosa*	1000–2000 mg/L (1–6 strains) [50]	4250 mg/L (multiple strains) [50]	/	Relevant MIC increase indicates resistance.
*Pseudomonas aeruginosa*	1000–2000 mg/L (1–6 strains) [50]	>2000 mg/L (*P. aeruginosa* PAO170 (1fabV)) [42]	Lack of precise MIC values, only information on a value higher than 2000 mg/L.	Relevant MIC increase can potentially indicate resistance, but there is insufficient data.
*Staphylococcus aureus*	0.5 mg/L (1635 strains) [49]	31 mg/L (*S. aureus* ATCC 6538 R1) [16]	/	Relevant MIC increase indicates resistance.
*Methicillin resistant Staphylococcus aureus* (MRSA)	0.5 mg/L (1635 strains) [49]	4 mg/L (MRSA F1855) [37]	Lack of data for MRSA strains.	Relevant MIC increase does not indicate resistance because of values only for *S. aureus*.
*Salmonella enterica*	8 mg/L (901 strains) [47]	0.25–4 mg/L (multiple strains) [40]	/	No relevant MIC increase does not indicate resistance.
*Salmonella enterica*	8 mg/L (901 strains) [47]	No MIC values. Only an increase of 1000 × mentioned (multiple strains) [39]	Lack of more accurate numerical MIC values.	Lack of MIC values does not provide sufficient data indicating relevance for resistance.
*Salmonella enterica*	8 mg/L (901 strains) [47]	64 mg/L *(S. enterica* L702) [97]	/	Relevant MIC increase indicates resistance.
Peracetic acid (0.2–3%) [98,99]				
*Pseudomonas aeruginosa*	100 mg/L (1 strain) [54]	400 mg/L (*P.aeruginosa* PAO1) [54]	Lack of sufficient MIC data and test sets to determine relevance.	Relevant MIC increase can potentially indicate resistance, but there is insufficient data.
*Bacillus atrophaeus* and *Escherichia coli*	1.25–5 mg/L in 3–6 mg/L (118 strains) [99,100]	MIC value 5 mg/L (multiple strains) (*B. atrophaeus* ATCC 9372) [99,100]	No MIC values in mg/L.	No relevant MIC increase does not indicate resistance.
Hydrogen peroxide (0.001–4%) [101,102,103]				
*Staphylococcus aureus*	0.2 mM–938 mg/L (2 strains) [103]	No MIC values 0.300 mg/L (*S. aureus* NCIMB 9518) [59]	No MIC values in mg/L.	Lack of MIC values does not provide sufficient data indicating relevance for resistance.
*Acinetobacter* spp.	47 mM–469 mg/L (48 strains) [57,101]	MIC value 13,000 mg/L (multiple strains) [57]	/	Relevant MIC increase indicates resistance.
*Bacillus* spp.	0.2 mM–1,875 mg/L (3 strains) [101,104]	MIC value 2 mM (*B. subtilis* PkatA:katX2) [104]	/	Relevant MIC increase indicates resistance.
Alcohols (70%) [87,101]				
*Enterococcus faecium* *Staphylococcus aureus*	Od 43,750 mg/L do 87,500 mg/L (7 strains) [101]	No MIC values. The only number of bacteria reduced in log^10^ [91]	No MIC values in mg/L.	Lack of MIC values does not provide sufficient data indicating relevance for resistance.
Aldehydes (0.5–4.0%) [105,106]				
*Salmonella enterica* (Formaldehyde)	0.3 mg/L (34 strains) [106]	0.125 mg/L (multiple strains) [90]	/	No relevant MIC increase does not indicate resistance.
*Pseudomonas aeruginosa* (Glutaraldehyde)	3750 mg/L (1 strain) [105]	No MIC values, only recognized increase by 3× *(P. aeruginosa* ATCC 15442) [93]	No sublethal MIC values and no MIC measurement units described.	Lack of MIC values does not provide sufficient data indicating relevance for resistance.
Chlorhexidine (0.5–4.0%) [107]				
*Vancomycin-resistant Enterococcus* (VRE)	4–16 mg/L (5 strains) [1]	8 mg/L (VRE VanA) [63]	/	No relevant MIC increase does not indicate resistance.
*Methicillin resistant Staphylococcus aureus* (MRSA)	8–128 mg/L (282 strains) [69]	Only MIC^50^ and MIC^90^ values <32 mg/L (multiple strains) [62]	No MIC values, only MIC^50^ and MIC^90^.	Lack of MIC values does not provide sufficient data indicating relevance for resistance.
*Enterococcus faecalis*	16 mg/L (56 strains) [96]	2500 mg/L (multiple strains) [66]	/	Relevant MIC increase indicates resistance.
*Enterococcus faecium*	32 mg/L (53 strains) [96]	250 mg/L (multiple strains) [66]	/	Relevant MIC increase indicates resistance.
*Pseudomonas aeruginosa*	8–64 mg/L (70 strains) [73,108]	70 mg/L *(P. aeruginosa* NCIMB 10421) [65]	/	Relevant MIC increase indicates resistance.
*Pseudomonas aeruginosa*	8–64 mg/L (70 strains) [73,108]	1024 mg/L *(P. aeruginosa* NCTC 6749) [64]	/	Relevant MIC increase indicates resistance.
Benzalkonium chloride and didecyldimonium chloride (0.01–5%) [109]				
*Pseudomonas* spp. (Benzalkonium chloride)	4–512 mg/L (11 strains) [77,110]	>350 mg/L *(P. aeruginosa* PA-29) [80]	/	No relevant MIC increase does not indicate resistance.
*Bacillus* spp. (Benzalkonium chloride)	16 mg/L (1 strains) [96]	140–160 mg/L *(B. cereus* ATCC 11778) [78]	Disinfectant concentration and disinfectant contact duration not provided.	Relevant MIC increase can potentially indicate resistance, but there is insufficient data.
*Staphylococcus aureus* (Benzalkonium chloride)	16 mg/L (1635 strains) [96] 0.25–4 mg/L (2 strains) [111]	32 mg/L (multiple strains) [79]	/	Relevant MIC increase indicates resistance.
*Listeria monocytogenes* (Benzalkonium chloride)	4–10 mg/L (31 strains) [111,112]	14 mg/L (multiple strains) [81]	/	Relevant MIC increase can potentially indicate resistance, although the MIC rise is minimal.
*Pseudomonas* spp. (Didecyldimonium chloride)	5–40 mg/L (11 strains) [113]	120 mg/L (multiple strains) [1]	/	Relevant MIC increase indicates resistance.

## Data Availability

Not applicable.
